# Clarifying the conflation of biochar carbon stability and its soil co-benefits

**DOI:** 10.1007/s42773-026-00581-4

**Published:** 2026-03-02

**Authors:** Robert W. Brown, David R. Chadwick, Davey L. Jones

**Affiliations:** School of Environmental and Natural Sciences, Bangor University, Deiniol Road, Bangor, LL57 2UW UK

**Keywords:** Environmental interaction, Durability, Carbon Markets, Agriculture

## Abstract

Not all biochar is equal. We clarify the frequent conflation between biochar carbon stability and soil co-benefits across research, policy, and markets. While stability ensures long-term carbon storage, co-benefits rely on more surface functionality from less stable biochar. Decoupling these dimensions enables designing biochar optimized for distinct functions.

## Highlights

- Biochar stability and soil benefits drive trade-offs in feedstock and pyrolysis conditions.
- Current reporting often conflates carbon durability with co-benefits, risking market misrepresentation.
- Clearer communication on biochar properties is vital to ensure credible carbon markets and policy decisions.

## Main

Greenhouse Gas Removal (GGR), specifically carbon dioxide removal (CDR), encompasses techniques that capture atmospheric greenhouse gases (GHGs) and store or chemically convert them with a degree of permanence. This approach is vital to achieving Net Zero and stabilising global temperature increases associated with anthropogenic GHG emissions and climate change, particularly for hard-to-abate sectors, such as agriculture and aviation (Workman et al. 2022). Biochar, the product of pyrolysis (exposure to high-temperature, low-oxygen conditions) of organic residues, is viewed as a promising and rapidly scalable CDR technology. Biochar-associated carbon credits currently account for a significant proportion of the voluntary carbon market, receiving only ~ 12% of CDR funding but accounting for 94% of delivered carbon credits globally in 2023 (CDR.fyi 2023).

Crop- and pastureland occupy ~ 40% of the global land surface (Winkler et al. 2021), offering an accessible sink for additional carbon storage, with application to the terrestrial land bank the most common end point for biochar storage. However, since the growing interest in biochar as a CDR technique, there has been significant conflation of site-specific biochar co-benefits in the scientific literature, which has subsequently fed through into wider practitioner, policy and market discussions.

Biochar can be broadly classified into two groups according to its application in soil; biochar specifically tailored for carbon stability (to maximise durability and make the biochar as recalcitrant and inert as possible), and biochar for use as a soil conditioner (to maximise the soil physicochemical benefits of biochar introduction). Each serves fundamentally different purposes.

**Fig. 1:**
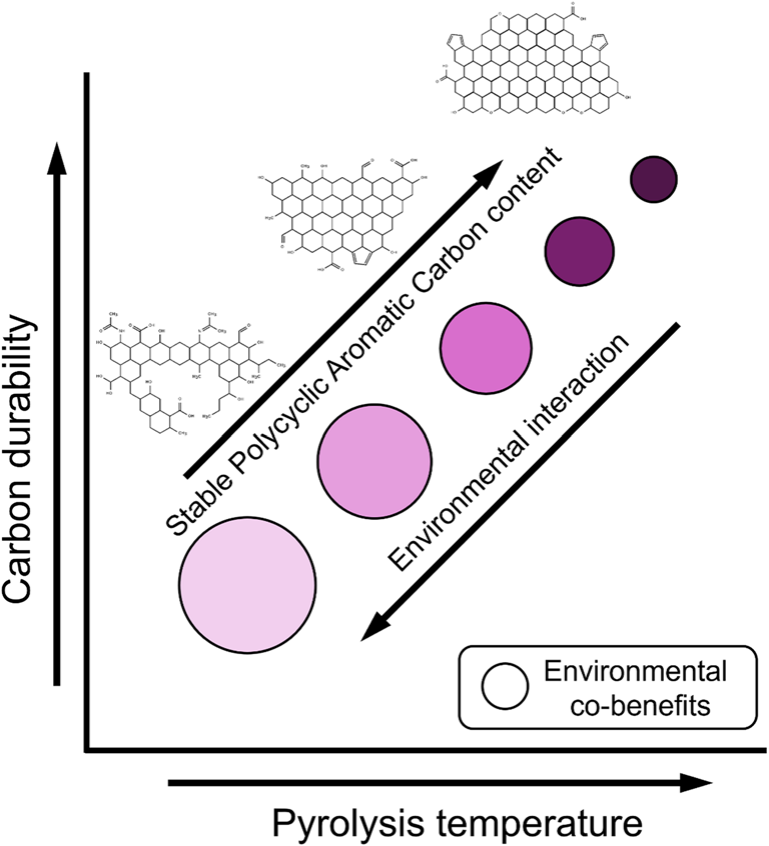
A conceptual diagram of the relationship between carbon durability, which is highly related to pyrolysis temperature, and the interaction with the soil environment

Feedstock composition and pyrolysis conditions fundamentally determine biochar properties, creating an inherent trade-off between carbon stability and co-benefits (Fig. 1). Higher production temperatures (> 700 °C) typically yield a higher density of stable polycyclic aromatic carbon structures (low initial atomic Hydrogen (H): Organic Carbon (C_org_) ratio) with greater long-term stability (residence times of > 1000 years; Ascough et al. 2019). However, with increasing temperature of pyrolysis (and therefore stability), there is a loss of surface functional groups (Janu et al. 2021) and therefore functional capacity (often indicated by high initial atomic Oxygen (O):C_org_ ratio; Bakshi et al. 2020), meaning reduced potential to contribute to nutrient and water retention and cation/anion exchange capacity. Thus, the more stable (or inert; Sanei et al. 2025) the biochar and its associated carbon, the less biotic and abiotic interaction with the surrounding environment.

Conversely, biochar produced at lower temperatures (350–500 °C) often retains more oxygen-containing functional groups beneficial for soil fertility (Bakshi et al. 2020) but may decompose more rapidly in aerobic soil environments (with a residence time of 100–300 years; Wang et al. 2016), reducing carbon sequestration potential (Adhikari et al. 2024). However, this biochar has more potential as a soil conditioner as well as the adsorption and stabilization of heavy metals and organic pollutants through surface functional groups, high porosity, and large specific surface area, thereby reducing their bioavailability and ecological risk (Liu et al. 2017). With significant research demonstrating the positive co-benefits of application (e.g., increased nutrient, water retention and pollutant attenuation (Hagemann et al. 2017; Acharya et al. 2024), particularly in laboratory settings and over short time periods. Equally, the persistence of co-benefits is rarely investigated, leaving the duration of their effects poorly understood. Because biochar has a finite number of surface bonding sites, understanding how long it takes for these sites to become saturated is essential for determining how often reapplication is needed.

As such, high-temperature pyrolysis of dense lignocellulosic material (i.e., woody biomass) remains the gold standard in durable biochar for carbon removal, but this is potentially not the case for the biochar-associated co-benefits. An exception to this is the burial of biochar in anaerobic, waterlogged soils where low temperature chars can maintain their functionality and maintain a high carbon storage potential (Rhymes et al. 2025).

Once biochar is applied to soil, there are very few methods to accurately quantify the contribution of biochar-derived organic carbon and soil organic carbon (SOC; Rathnayake et al. 2024). However, evidence suggests that biochar can induce further carbon storage through negative priming and immobilization (Tang et al. 2025), leading to an increase in the total organic carbon pool (Gross et al. 2024). Although few studies have examined the impact of biochar stability on soil carbon accrual, particularly in the field over long timescales, it is also important to differentiate between highly recalcitrant forms of carbon (i.e. biochar-associated) and other native pools of soil organic matter, which may be actively cycled over shorter timeframes, and keeps soil naturally fertile (Giannetta et al. 2023).

However, all current biochar carbon crediting options rely on modelling to predict carbon durability, using the best available science. Most commonly, the initial atomic H:C ratio is used as a relatively low-cost and widely available technique. However, emerging techniques include using geological proxies for stability (e.g. the inertinite benchmark; Sanei et al. 2025) and more advanced chemical proxies (e.g., the Hydrogen Pyrolysis (HyPy) derived Stable Polycyclic Aromatic Carbon (SPAC) fraction (Howell et al. 2022)). While no study has directly compared an exhaustive number of biochar stability analysis methods, recent reviews by Adhikari et al. (2024) and Leng et al. (2019) give a broad summary. There is strong evidence that low atomic H:C ratios are consistently related to higher biochar carbon stability and therefore durability in the environment (Azzi et al. 2024; Adhikari et al. 2024). However, researchers often fail to report key variables such as the biochar feedstock, pyrolysis condition, or physicochemical properties (particularly the atomic H:C and O:C ratios) of the resulting char, often presenting findings on agricultural benefits alongside carbon sequestration potential without clarifying the inherent trade-offs between these objectives. Accordingly, the environmental additionality associated with biochar application may be reduced when using high stability biochar.

Additionally, it may be possible to increase the biochar-associated co-benefits from high stability biochar using a number of methods. For example, biological colonisation of high-stability biochar can be enhanced through surface activation using compost, slurry, or manure (Gao et al. 2023; Jaufmann et al. 2024), as well as through the formation of biochar–fertiliser composites (Melo et al. 2022) and biochar–microbe inoculants (Egamberdieva et al. 2018). These treatments introduce nutrients, organic acids, and microbial communities that modify the biochar surface, potentially adding functional groups and biofilms that provide a physical scaffold for microbial adhesion and nutrient exchange. Even though high-temperature biochar is chemically inert, its high porosity and surface area provide an excellent physical scaffold for microbial communities once nutrient-rich materials are introduced.

Recent work shows that different activation approaches vary in both effectiveness and practical constraints. Co-composting or coating biochar with compost or manure can substantially enhance microbial colonization and nutrient retention, but outcomes depend on compost quality and can sometimes trigger short-term shifts in nitrogen dynamics or mild priming effects (Abban-Baidoo et al. 2024; Guo et al. 2020). Biochar–fertiliser composites improve nutrient use efficiency and can provide slow-release benefits, though they require extra processing and may increase production costs (Rubel and Wei 2025). Similarly, using biochar as a carrier for microbial inoculants can improve the survival and function of beneficial microbes, but success depends on matching inoculant strains with biochar properties and soil conditions (Bolan et al. 2023). Overall, these methods are promising but should be selected with consideration of their trade-offs, site conditions, and the specific co-benefits being targeted.

It is also important to note that the impact of biochar application on co-benefit delivery is highly dependent on the condition and environment that the biochar is being applied into. In initially productive soils, often in temperate climates, where fertility and fertilizer inputs and buffering capacity are high, it is less likely that significant gains in productivity through biochar application will occur (Lévesque et al. 2022; Jeffery et al 2017). Whereas highly weathered soils in the tropics and degraded soils may experience greater increases in productivity, as any additional gains through liming, and potentially nutrient retention are likely to have significant impacts on yields (Basak et al. 2022). But this is not a reason to avoid creating and applying biochar in temperate climates; efforts should be made to store carbon in all climates and soils. However, we advocate tailoring biochar to maximize the desired direct and co-benefits in the specific end use case i.e., “designer biochar” (Zhang et al. 2022; Zhou et al. 2024).

Moving forward, much greater clarity is required throughout biochar research. Ambiguity has the potential to propagate from the site- and feedstock-specific contexts, which result in significant co-benefits of biochar application, through to wider (generic) policy discussions and markets, potentially creating confusion about the benefits specific biochar products can realistically deliver. This is especially true at realistic loading rates and under field conditions. It is imperative to highlight the fact that not all biochars are created equal and that divergent feedstock and pyrolysis conditions can result in vastly different biochar batch properties. Likewise, not all soil contexts respond similarly, broadly speaking, degraded or tropical soils benefit more from the wider co-benefits of biochar application than non-degraded or temperate soils (Jeffery et al. 2017).

Failing to communicate the nuance that not all biochar is equal, results in potential conflation of biochar’s carbon stability and its soil co-benefits, particularly in cases where biochar is not activated or co-applied with organic mixes (e.g., composts). There is a risk of undermining confidence in carbon removal markets through overstating the co-benefits and environmental additionality. As the voluntary carbon market continues to grow and biochar occupies an increasingly prominent position within it, transparent communication about the capabilities and limitations of different biochar products is essential for the integrity of the carbon market. Without such transparency, oversimplification could lead to a misallocation of resources in our efforts to address climate change.

## Data Availability

Data sharing not applicable—no new data generated.
